# P-wave oversensing during atrial tachycardia in patients with an extravascular ICD

**DOI:** 10.1093/ehjcr/ytaf537

**Published:** 2025-10-16

**Authors:** Alexander Breitenstein

**Affiliations:** Department of Cardiology, University Heart Center, University Hospital Zurich, Raemistrasse 100, Zurich 8091, Switzerland

## Case description

The first patient, a 54-year-old woman with hypertrophic cardiomyopathy, received an EV-ICD for primary prevention. The second patient (48-year-old man) experienced an out-of-hospital cardiac arrest due to ventricular fibrillation without an identifiable reversible cause. In both cases, implantation was uneventful, and initial sensing parameters showed good ventricular signal amplitude with no significant *P*-wave oversensing during sinus rhythm. In both patients, the sensing vector was Ring1-Ring2 (near-field sensing between the sensing poles), with a ventricular sensitivity of 0.15 mV (standard recommendation). The oversensing prevention was set to level 4 (on a scale from 1 to 6, where 1 is the most sensitive and 6 the least sensitive).

**Figure 1:**
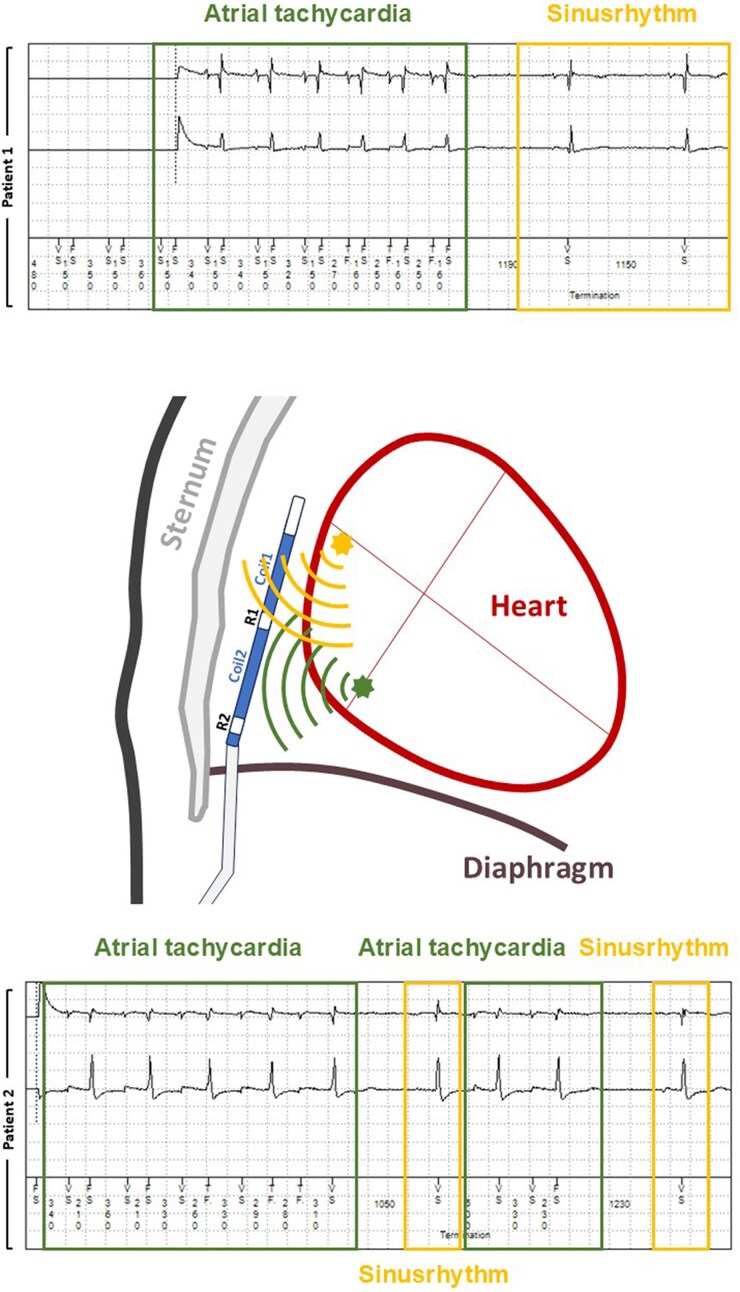
Arrhythmia recording of patient 1 (upper panel) and patient 2 (lower panel) during brief episodes of atrial tachycardia with subsequent P-wave oversensing, which is not present during sinus rhythm.

However, during follow-up, remote transmissions revealed *P*-wave oversensing during brief episodes of atrial tachycardia in both patients (*Figure 1*). These episodes were associated with altered atrial electrical activation resulting in increased *P*-wave amplitude and changed morphology which is a known phenomenon for atrial tachycardias,^1^ leading to oversensing by the device. Although the episodes were self-limiting and did not trigger inappropriate therapy, they highlight a potential risk for signal oversensing. If such arrhythmias are longer-lasting, it may be useful to investigate for potential atrial oversensing during episodes of tachycardia. Due to the short and self-limiting character of these episodes in both patients, no change in device settings were necessary. However, adjusting the sensing vector or sensitivity settings may mitigate this issue. Similar to transvenous defibrillators, the sensitivity in the substernal ICD is dynamic and increases with time after the last ventricular event. For the first period, after a blanking phase, the oversensing prevention feature regulates the sensitivity of the device for a certain time period (on a scale from 1 to 6, where 1 is the most sensitive and 6 the least sensitive) followed by the baseline sensitivity (which is nominally set to 0.15 mV). Additionally, advanced device algorithms such as the ‘Smart Sense’ enhances the discrimination between true ventricular arrhythmias and *P*-wave oversensing (The ‘Smart Sense’ algorithm is a Medtronic specific feature which detects alternating low (*P*-wave) and high (R-wave) amplitude signals together with a QRS farfield morphology which is similar to normal sinus rhythm).^2^

**Consent:** Both patients have given written informed consent (compliant with COPE guidelines).

## Data Availability

The data underlying this article cannot be shared publicly due to the privacy of the individuals that participated in the study. The data will be shared on reasonable request to the corresponding author.
